# Correction

**Published:** 1885-05

**Authors:** 


CORRECTION.—On page 172, first line, for "Nokitansky" read "*Rokitansky;*" ninth line for "ten" per cent, read "*two*" per cent. Page 173, fourth line, for "brought" a cure, read "*wrought*" a cure.

